# Immune Amplification of Murine CD8^+^ Suppressor T Cells Induced via An Immune-Privileged Site: Quantifying Suppressor T Cells Functionally

**DOI:** 10.1371/journal.pone.0022496

**Published:** 2011-08-02

**Authors:** Roshanak Sharafieh, Yen Lemire, Sabrina Powell, James O'Rourke, Robert E. Cone

**Affiliations:** Department of Immunology, University of Connecticut Health Center, Farmington, Connecticut, United States of America; Saint Louis University School of Medicine, United States of America

## Abstract

**Background:**

CD8^+^ suppressor T cells exert antigen-specific suppression of the expression of hypersensitivity by activated T cells. Therefore, CD8^+^ suppressor T cells serve a major regulatory role for the control of active immunity. Accordingly, the number and/or activity of CD8^+^ suppressor T cells should be influenced by an immune response to the antigen. To test this hypothesis we used an adoptive transfer assay that measures the suppression of the expression of delayed-type hypersensitivity (DTH) by CD8^+^ suppressor T cells to quantify the antigen-specific suppression of DTH by these suppressor T cells.

**Methods:**

Suppressor T cells were induced in the spleens of mice by the injection of antigen into the anterior chamber of an eye. Following this injection, the mice were immunized by the same antigen injected into the anterior chamber. Spleen cells recovered from these mice (AC-SPL cells) were titrated in an adoptive transfer assay to determine the number of AC-SPL cells required to effect a 50% reduction of antigen-induced swelling (Sw50) in the footpad of immunized mice challenged by antigen.

**Results:**

Suppression of the expression of DTH is proportional to the number of AC-SPL cells injected into the site challenged by antigen. The number of AC-SPL cells required for a 50% reduction in DTH-induced swelling is reduced by injecting a cell population enriched for CD8^+^ AC-SPL cells. Immunizing the mice receiving intracameral antigen to the same antigen decreases the RSw50 of AC-SPL cells required to inhibit the expression of DTH.

**Conclusions:**

The results provide the first quantitative demonstration that the numbers of antigen-specific splenic CD8^+^ suppressor T cells are specifically amplified by antigen during an immune response.

## Introduction

CD8^+^ suppressor T cells execute a Qa-1-restricted, antigen-specific suppression of activated T cells [1]–[12]. Unlike CD4^+^, FoxP3^+^ regulatory T cells (T reg), CD8^+^ suppressor T cells inhibit activated T effector cells whose actions may cause autoimmune disease [1]–[4]. Accordingly, understanding the activation and activity of these suppressor T cells could provide insight into the mechanisms of suppression and perhaps the development of therapeutic modalities that use these cells to modulate inflammatory diseases. Surprisingly, although known for more than 25 years [1], [12], the activation, indeed the existence of CD8^+^ suppressor T cells is not well understood.

The activity of CD8^+^ suppressor T cells is evident after immunization [3], [5], consistent with the antigen specificity of the suppression mediated by these cells. Accordingly, immunization should induce a specific increase in CD8^+^ suppressor T cells responding to the antigen and/or a variable region epitope of the receptor of T cells specific for the antigen [1], [2], [8]. This increase in suppressor T cells could be due to the amplification of T cell clones that are the precursors of CD8^+^ suppressor T cells and/or to effector T cells that are “converted” to a suppressive phenotype [10], [11]. Although CD8^+^ suppressor T cells are phenotypically and functionally distinct from CD4^+^, FoxP3^+^ regulatory T cells there is presently no marker to identify (and therefore enumerate) these cells. Accordingly, a quantitative functional assay of the activity of suppressor T cells could provide a metric to estimate CD8^+^ suppressor T cells and understand the nature of the activation and function of these immunoregulatory T cells *in vivo*.

Although there are several procedures to induce suppressor T cells *in vivo* [1], the injection of antigen into the anterior chamber of an eye attracts circulating F4/80^+^ monocytes to the anterior chamber that traverse the anterior chamber and recirculate to the thymus and spleen [13]. In the spleen, these monocytes interact with CD4^+^ T cells, B cells and recruited regulatory recent thymic emigrants to induce antigen specific CD8^+^ T suppressor T cells that suppress the expression of DTH to the same antigen injected into the anterior chamber [13]–[21]. In aggregate, Anterior Chamber Associated Immune Deviation (ACAID) in rodents appears to be the result of a moderate inflammatory response to ocular injury. Quantifying the functional activity of splenic suppressor T cells induced by the intracameral injection of antigen could provide a metric to quantify ACAID) and provide information to quantify this phenomenon as well as understand the development of suppressor T cells.

We here investigated the immunologic basis of the *in vivo* production of splenic antigen-specific suppressor T cells induced by the intracameral injection of antigen using an *in vivo* assay of the suppression of the expression of DTH by CD8^+^ suppressor T cells. The results show for the first time that immunization amplifies quantitatively splenic CD8^+^ suppressor T cells induced by the intracameral injection of antigen. Moreover, the suppressive activity of these cells is transient and only expressed for less than three days at the site in immunized mice where the suppressor cells were introduced.

## Materials and Methods

### Mice

Female or male BALB/c or C57Bl/6 mice, 8–10 weeks old, were purchased from Charles River/NCI Laboratories (Wilimington,MA). The mice were maintained in the Center for Laboratory Animal Care of the University of Connecticut Health Center. All work with animals was approved previously by the University of Connecticut Health Center Animal Care Committee (ACC-2004-098, 2007-369). All animals were treated according to the ARVO Statement for the Use of Animals in Ophthalmic and Vision Research.

### Reagents and Immunization

2,4,6, Trinitrobenzene sulphonic acid (TNBS), bovine serum albumin (BSA) and ovalbumin (OVA) were purchased from Sigma Aldrich Chemical Co. (St. Louis, MO). Trinitrophenylated bovine serum albumin (TNP-BSA) was prepared by conjugating TNBS to BSA as described [9]. Picryl chloride (PCl), 2-chloro-1,3,5-trinitorobenzene was purchased as 2-chloro-5-trypthtane from Chemical Alta Ltd (Edmonton, Alberta, Canada). Unless specified otherwise, mice were immunized by a single sc injection of 200 µg TNP-BSA or OVA in 50 µl Freund's Complete Adjuvant (CFA, Sigma) into a flank. In general, the mice were challenged to induce the expression of DTH sensitivity 7–10 days after the mice were immunized.

### Initiation of the expression of Delayed-Type Hypersensitivity (DTH)

Contact sensitivity (CS) to TNP in TNP-BSA-sensitized or naïve mice was induced by the epicutaneous application of 15 µl 1% PCl (the antigenic equivalent of TNP) in acetone∶olive oil 4∶1 to a footpad. The CS response was usually measured approximately 24 hr and 48 hr after the application of PCl by measuring footpad thickness with an engineer's digital micrometer (Mitatoyo, Tokyo, Japan). Mice were anesthetized with ketamine/xylazine (please see below) and the thickness of each footpad measured. One footpad was then challenged with PCl and the other with vehicle only. Twenty-four hr later the mice were anesthetized again and the thickness of each footpad measured. The thickness of each footpad before the application of PCl was subtracted from that of the thickness of the footpad 24–48 hr after the application of PCl. Swelling was computed as the difference between the thickness of the challenged footpad minus that of the unchallenged footpad.

### Injection of antigen into the anterior chamber (intracameral injection)

Mice were immunized with TNP-BSA 7 days after the injection of antigen into the anterior chamber as described [9]. Mice were anesthetized with ketamine (75 mg/kg)/xylazine (15 mg/kg) and under a dissecting microscope a 30 g needle attached to a cannula attached to a manually controlled Hamilton syringe (Stoelting Co, Woodale, IL) was inserted into the anterior chamber. Approximately 4 µl phosphate-buffered saline (PBS pH 7.2) containing 4 µg TNP-BSA or 50 µg OVA was injected into the anterior chamber (AC). The mice recovered approximately 30 min after the injection and exhibited no distress, eating and drinking normally.

### Spleen cell preparation

Seven days after receiving an intracameral injection of TNP-BSA or OVA, the mice were euthanized, spleens recovered from the mice, diced and expressed through a 70 µm cell strainer (Becton Dickinson, BD, Rockville,MD), into PBS. These spleen cells (AC-SPL cells) were washed 2 times. To separate the AC-SPL cells based on the expression of CD8, the cells were suspended in PBS or BD™ IMag separation buffer ). The spleen cells were separated by immunomagnetic beads into CD8^+^ and CD8^−^ populations with a BD™ CD8 T lymphocyte enrichment set according to the manufacturer's protocol. Enrichment was assessed by immnofluorescent staining of the cells using BD™ fluorescein isothiocyanate (FITC)-anti-CD8 antibodies. Approximately 80–95% CD8^+^ spleen cells were prepared after treatment with the CD8 enrichment kit.

### Adoptive transfer (Local Transfer of Suppression, LTS)

Splenic regulatory T cells: Regulatory-effector T cells were assayed by the Local Transfer of Suppression assay (LTS [9]) for CD8^+^ regulatory T cells. All cells were suspended in PBS for injection. Cells were quantified with a Beckman Coulter Counter. Between 250 and 25000 spleen cells from AC-injected donors at 1×10^8^ /ml were injected subcutaneously (sc) into the footpads of immunized mice immediately following epicutaneous challenge with PCl. Swelling induced by PCl was measured 24–48 hr after challenge.

### Statistics

Statistical significance was calculated by one-way ANOVA. P-values were determined by the Student-Neuman-Keuls test and p values<0.05 were considered significant.

## Results

### Suppression of the expression of DTH in the LTS by AC-SPL cells is proportional to the number of transferred cells

**Figure 1:**
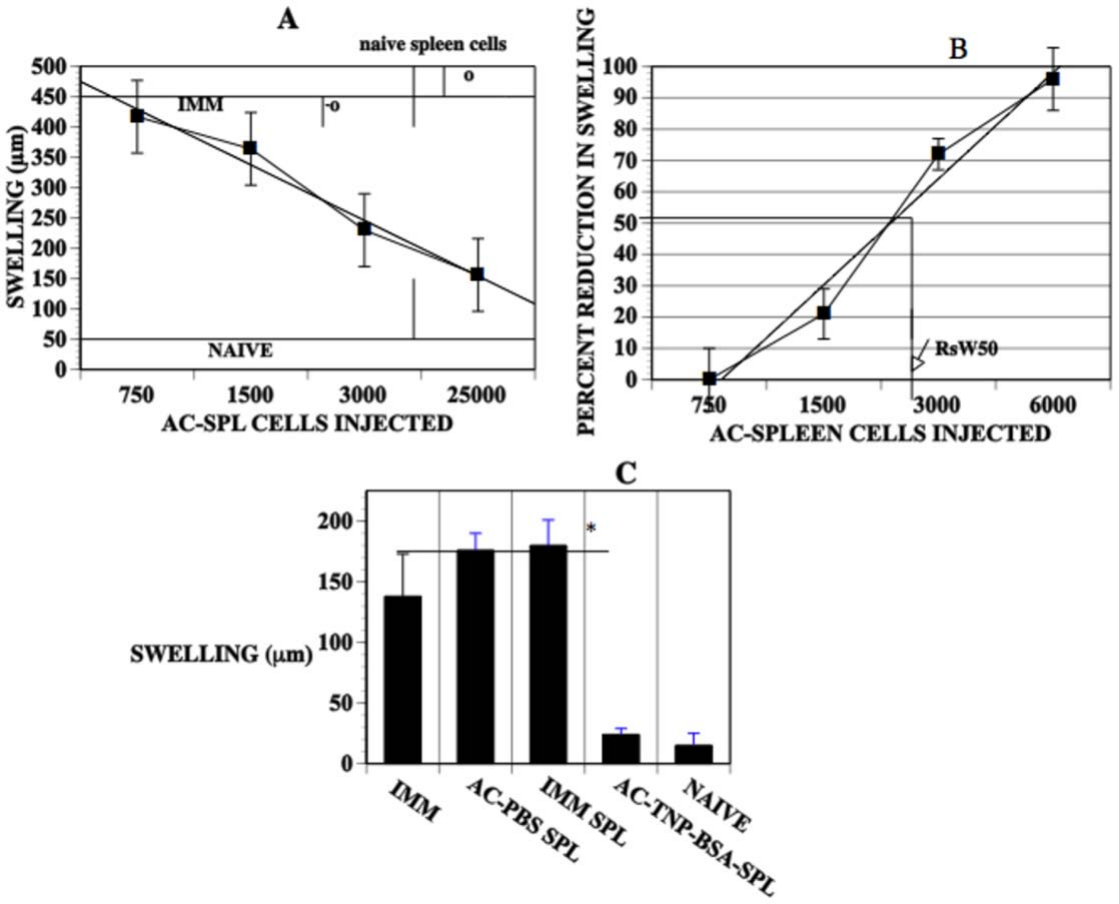
The suppression of the expression of DTH in the LTS by intracamerally-induced AC-SPL spleen cells is proportional to the number of transferred AC-SPL cells. (A) Reduction in antigen-induced swelling by AC-SPL cells is proportional to the number of AC-SPL cells transferred to the antigen challenge site. Spleen cells were recovered from donors that received an intracameral injection of TNP-BSA and were immunized to TNP-BSA. Unfractionated AC-SPL cells were quantified with a Coulter counter and the number of cells shown injected id into the footpads of TNP-BSA-immunized recipients immediately after the footpads were challenged with epicutaneous PCl. Open circles show naïve spleen cells. Swelling was measured 24 hr after challenge. The data is pooled from 3–4 separate experiments and is the mean swelling +/− SE of 8–12 mice/group. IMM: mean swelling of immunized mice that received no AC-SPL cells. NAÏVE: mean swelling of challenged, non-immunized mice. The straight line was generated by curve fit software. (B) The metric (RSw50) quantifies the suppression of DTH-induced swelling by AC-SPL cells. Suppression of DTH-induced swelling by AC-SPL cells was computed as the specific swelling (µm) of the challenged footpad receiving AC-SPL cells/ specific swelling of the challenged footpad that did not receive AC-SPL cells X 100. The straight line was generated by curve fit software. The data represents the pooled mean suppression of swelling +/− the standard error of the mean of five separate experiments, 15 mice/group. The number of AC-SPL cells providing 50% suppression of DTH swelling (RSw50) is shown and was computed based on the curve fit. (C) Only AC-SPL cells recovered from mice receiving an intracameral injection of TNP-BSA suppress TNP-induced swelling in TNP-immunized mice. The footpads of mice immunized with TNP-BSA (IMM) or naïve mice received 25,000 spleen cells recovered from TNP-BSA-immunized mice (IMM-SPL) TNP-BSA-immunized that received intracameral TNP-BSA (AC-TNP-BSA-SPL) or PBS only (AC-PBS-SPL) concomitant with epicutanteous PCl. Swelling was measured before and 24 hr after challenge. Data is the mean swelling in three experiments, 3–4 mice/group/experiment (total of 9–12 mice/group). * P<.01.

AC-SPL cell populations contain CD8^+^ suppressor T cells that suppress the expression of DTH when transferred to a site in immunized mice challenged with antigen to induce a DTH reaction [9], [21]. Presently, there is no marker to identify these suppressor cells so that they can be quantified. We reasoned that we could quantify the activity of CD8^+^ suppressor T cells by measuring the suppression of DTH using an assay that measures solely CD8^+^ suppressor T cell-mediated suppression of the expression of DTH [9], [21]. Therefore, suppressor cell donors received an intracameral injection of TNP-BSA and were immunized systemically with TNP-BSA to generate CD8^+^ suppressor T cells. One week after these mice were immunized, spleens were recovered and spleen cell suspensions (AC-SPL cells) prepared. We then titrated the activity of the suppressor cells to suppress the expression of DTH in immunized mice. Two hundred fifty-25, 000 total spleen cells recovered from the donors that received an intracameral injection of antigen (AC-SPL cells ) were injected into the footpads of the TNP-BSA-immunized mice immediately after the footpad was challenged epicutaneously with PCl. Positive control immunized mice received epicutaneous PCl but no AC-SPL cells and negative controls were naïve mice challenged with epicutaneous PCl only. The thickness of the footpads was measured before the challenge with antigen, +/− AC-SPL cells and then 24 and 48 hr after challenge with PCl to determine specific footpad swelling. Immunized mice that did not receive AC-SPL cells had robust footpad swelling in response to the antigenic challenge (greater than 2-fold the thickness obtained with naïve mice). The PCl-induced swelling of the footpads of TNP-BSA-immunized mice that received AC-SPL cells was reduced significantly (Fig. 1 A–C). There was a linear relationship between the reduction in the elicited footpad swelling and the number of AC-SPL spleen cells injected into the footpad. (Fig. 1A). Twenty-five thousand or 2800 naïve spleen cells did not suppress the expression of DTH. Spleen cells recovered from control TNP-BSA-immunized mice that did not receive an intracameral injection of TNP-BSA or received an intracameral injection of PBS only did not suppress swelling (Fig. 1C). Because of the linearity in the reduction of PCl-induced swelling versus the number of AC-SPL cells injected into the challenge site, we derived a metric for the percent reduction in reduction in swelling proportional to the number of AC-SPL spleen cells injected with challenge antigen using a curve fit generated by the percent reduction in DTH-induced swelling vs the number of regulatory spleen cells injected into the site challenged by antigen. The derivation of this metric is shown in Fig. 1B. Approximately 2,800 AC-SPL cells injected into the challenge site produced a 50% reduction in swelling termed RSw50 since the 50% reduction in swelling falls on the curve fit plot (Fig. 1B).

**Figure 2:**
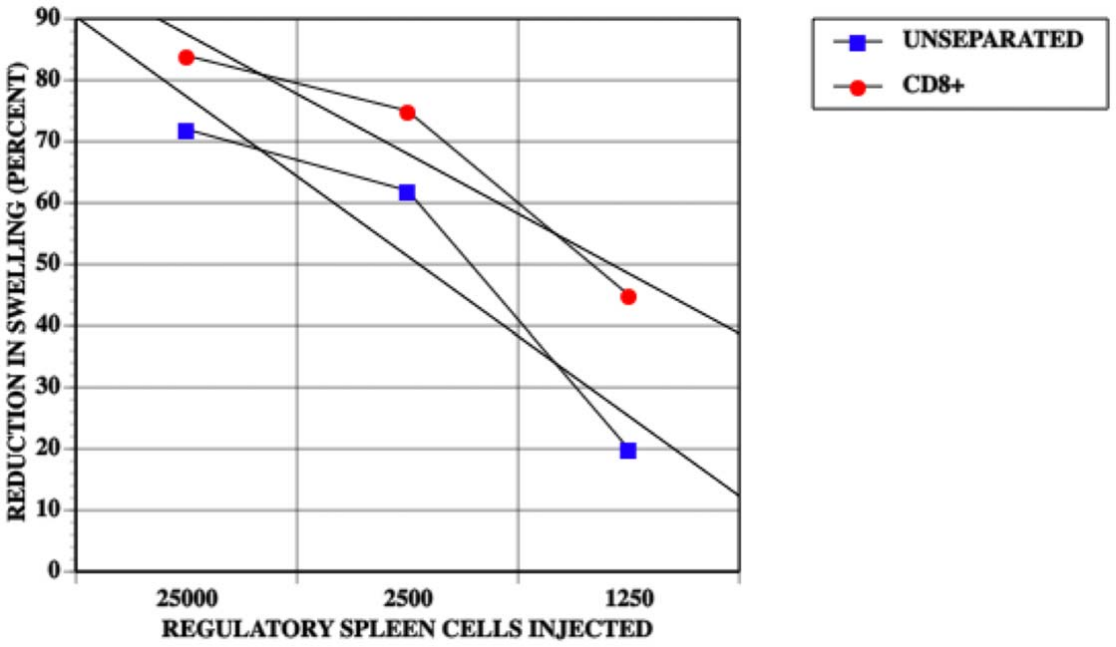
The RsW50 of regulatory spleen cells is decreased by enriching for CD8^+^ regulatory spleen cells. The footpads of mice immunized with TNP-BSA and CFA 9 days previously received id CD8^+^ cells or unseparated AC-SPL cells prepared as in Fig. 1 immediately after the footpad was challenged with epicutaneous PCl. Swelling was measured 24 hr later. The data is pooled from two separate experiments with six mice /group. NAÏVE: non-immunized mice, IMM: immunized mice that did not receive regulatory spleen cells, CD8^+^: immunized mice that received CD8^+^ regulatory spleen cells. p<0.01. Straight lines were generated by curve fit software.

Because CD8^+^ splenic suppressor T cells induced by the intracameral injection of antigen are solely responsible for the reduction in antigen-induced swelling in the LTS, we compared quantitatively the suppressive activity of unseparated AC-SPL cells to that of AC-SPL cells enriched for CD8^+^cells. CD8^+^ spleen cells recovered from mice receiving intracameral TNP-BSA were enriched to 85–95% from approximately 33–35% CD8^+^cells. (data not shown). Graded numbers of unseparated and CD8^+^ spleen cells were injected into the footpads of TNP-BSA-immunized mice immediately after the footpads were challenged with epicutaneous PCl. The RSw50 obtained with unseparated AC-SPL cells was approximately 2500 and 1260 for CD8^+^ AC-SPL cells (Fig. 2). Therefore, the number of AC-SPL cells enriched for CD8^+^ cells needed to cause a 50% reduction in antigen-induced footpad swelling is approximately half that required using unseparated spleen cells suggesting at least a 2-fold enrichment in the CD8^+^ AC-SPL suppressor cells. The injection of CD8^−^spleen cells recovered from mice receiving an intracameral injection of TNP-BSA did not suppress footpad swelling (data not shown).

### Immunization amplifies splenic suppressor T cells induced by the intracameral injection of antigen

**Figure 3:**
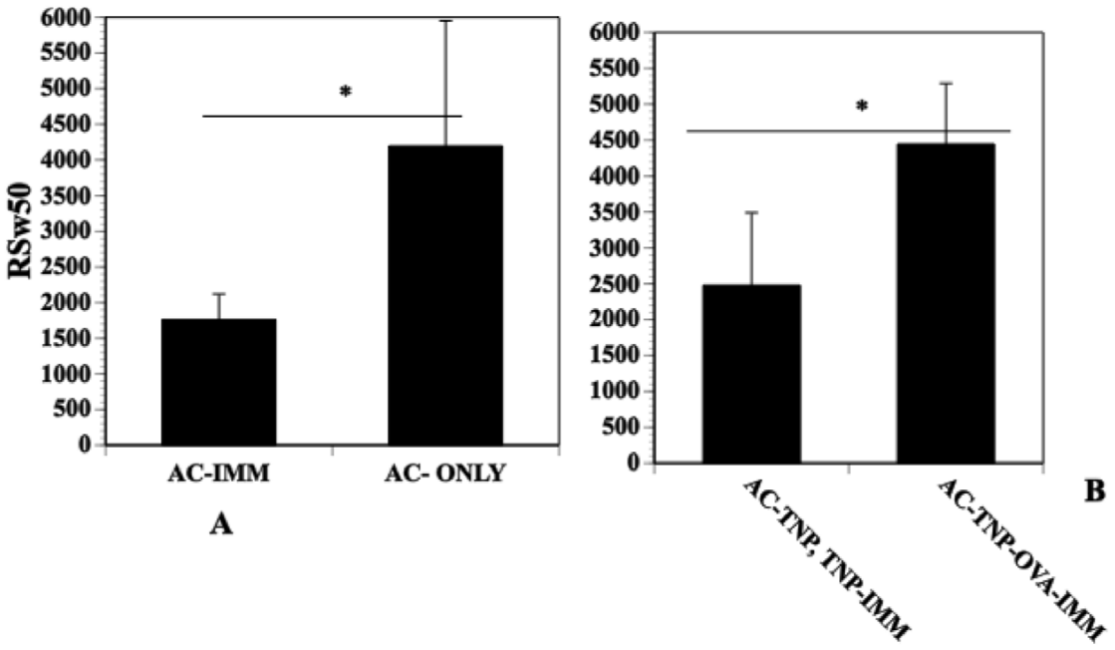
Immunization reduces the RSw50 of AC-SPL cells. (A) BALB/c mice received an injection of TNP-BSA into the anterior chamber. Seven days after receiving an intracameral injection of TNP-BSA, some mice were immunized with TNP-BSA/CFA. Spleen cells were recovered from the immunized, AC-injected mice (AC-IMM) and mice that received an injection of antigen into the AC only (AC-ONLY) one week after the immunization of AC-injected mice or one week after intracameral injection only. Twenty-five thousand AC-SPL cells were injected into the footpads of TNP-BSA-immunized mice immediately after the footpads were challenged with epicutaneous PCl. (B) Immunization-induced increase in suppression is antigen-specific. Seven days after mice received an intracameral injection of TNP-BSA, the mice were immunized with TNP (AC-TNP), TNP-IMM or OVA (AC-TNP-OVA-IMM). Seven days after immunization, spleens were recovered from the mice and recovered spleen cells injected into the footpad of TNP-BSA-immunized mice immediately after the footpad was challenge with epicutaneous PCl. Swelling was measured 24 hr later. The data represents the mean RsW50 +/− the standard error of the mean for three experiments, 9 mice/group. *p<0.02.

To determine whether immunizing amplifies splenic AC-SPL suppressor cells, mice were immunized with TNP-BSA 7 days after the mice received an intracameral injection of TNP-BSA. AC-SPL cells recovered from these mice were compared for the suppression of the expression of DTH to naïve mice that received an intracameral injection of TNP-BSA only. The RSw50 obtained from AC-SPL cells recovered from immunized mice, was significantly less than that of AC-SPL cells recovered from mice receiving an intracameral injection of TNP-BSA only (Fig. 3A) indicating an increase in number and/or activity of suppressor cells. To determine the antigen specificity of this increase, mice receiving an intracameral injection of TNP-BSA were subsequently immunized with TNP-BSA or OVA. One week after immunization, spleen cells were recovered and titrated in the LTS assay for suppressor cells that regulated the DTH reaction to PCl. Approximately 2500 spleen cells recovered from mice that received an intracameral injection of TNP-BSA and were immunized with TNP-BSA suppressed footpad swelling by 50% in TNP-BSA- immunized mice. Comparatively, forty five hundred spleen cells recovered from mice receiving an intracameral injection of TNP-BSA and immunization with OVA suppressed PCl-induced swelling in TNP-BSA-immunized mice by 50% (Fig. 3B).

### Quantitative increase of suppressor AC-SPL cells that suppress specifically the expression of DTH depends on antigen dose

**Figure 4:**
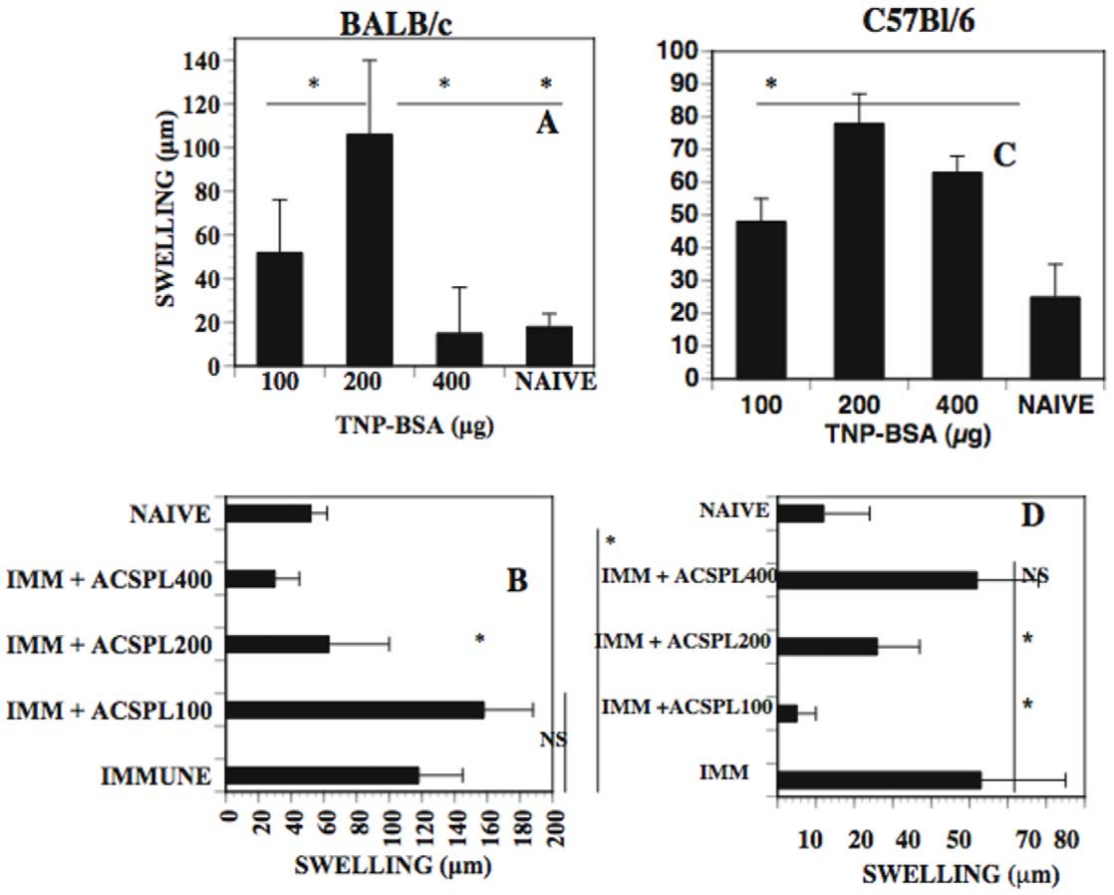
The expansion of AC-SPL suppressor cells that suppress the expression of DTH is strain and antigen dose -dependent. Seven days after mice were immunized with TNP-BSA, a footpad was challenged with epicutaneous PCl. Footpad thickness was measured before and 24 hr after challenge. Five thousand AC-SPL cells were injected into a footpad of mice immunized with TNP-BSA immediately after the footpad was challenged with epicutaneous PCl. Footpad thickness was measured before and 24 hr after challenge and swelling computed. (A): Effect of immunizing dose of antigen on DTH-induced swelling in BALB/c (A) C57BL/6 mice (C), Generation of suppressive AC-SPL cells is antigen dose dependent (B): BALB/c, D: C57BL/6. Data represents the mean swelling +/− S.E.M. of 12 mice/group , 3 experiments. * p<0.05,NS: not significant.

The preceding results demonstrate that immunization amplifies quantitatively splenic suppressor T cells active in the LTS. Accordingly, the dose of antigen used in an immunization should influence the propagation of these cells. To investigate this hypothesis, BALB/c or C57BL/6 mice received an intracameral injection of TNP-BSA and were then immunized with different amounts of TNP-BSA. One week after immunization, spleen cells were recovered from the mice. To ensure quantitative sensitivity in the LTS, a limiting number (5000) of AC-SPL cells that falls on the linear titration curve (fig. 1) were injected into the footpads of syngeneic TNP-BSA-immunized mice immediately after the footpad was challenged with epicutaneous PCl to elicit the expression of DTH. Figure 4 compares the dose of TNP-BSA required to stimulate DTH in BALB/c or C57BL/6 mice (Fig. 4 A,C) and the dose of immunizing TNP-BSA optimal to expand splenic suppressor T cells after the intracameral injection of TNP-BSA (Fig. 4 B,D). Two hundred µg of TNP-BSA was optimal to induce DTH to TNP in both C57BL/6 and BALB/c mice However, 100 or 400 µg TNP-BSA were less effective at inducing DTH in BALB/c mice. Suppressor spleen cells recovered from C57BL/6 donors immunized with 100 µg TNP-BSA were most effective in suppressing the expression of DTH in the LTS (Fig. 4D). Suppressor spleen cells recovered from C57BL/6 mice immunized with 400 µg TNP-BSA were not suppressive. However, the 400 µg dose was optimal to expand suppressor spleen cells in BALB/c mice that received an intracameral injection of TNP-BSA. Spleen cells recovered from BALB/c mice immunized with 400 µg TNP-BSA but did not receive an intracameral injection of TNP-BSA did not suppress PCl-induced swelling (data not shown).

### The suppression of the expression of DTH by CD8^+^ regulatory spleen cells is transient

**Figure 5:**
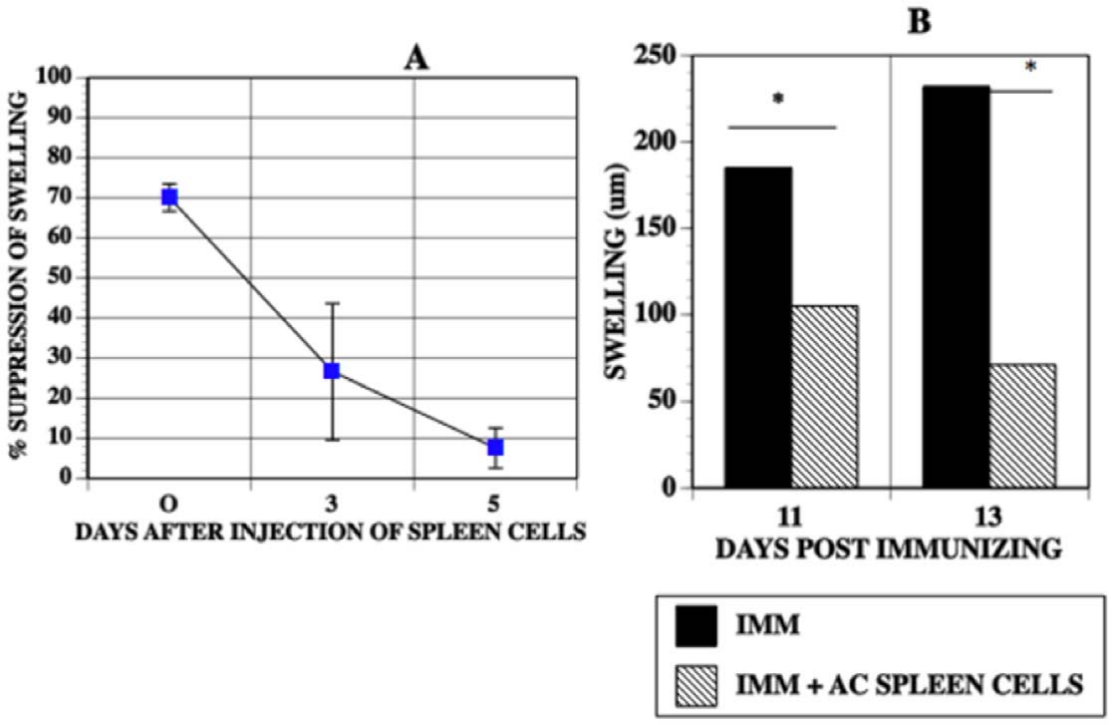
Durability of cell-mediated suppression OF DTH-mediated swelling. The footpads of mice immunized with TNP-BSA were injected with 25000 spleen cells recovered from TNP-BSA-immunized mice that received an intracameral injection of TNP-BSA. The footpads were challenged with epicutaneous PCl immediately after receiving the spleen cells 0, 3 or 5 days after receiving the spleen cells. Footpad thickness was measured 24 and 48 hr after challenge with PCl. (A) Percent suppression is calculated as : 100- swelling (µm) immunized mice+regulatory spleen cells. Swelling (µm) immunized only. Data represents mean suppression +/− S.E.M. 9 mice/group, 3 experiments. (B) The footpads of mice immunized 11 or 13 days previously with TNP-BSA received id 25000 spleen cells recovered from TNP-BSA-immunized mice that received an intracameral injection of TNP-BSA and epicutaneous PCl. Swelling was measured 24 and 48 hr after challenge. Data shows the mean swelling (µm) of 8 mice/group, two experiments. * p<0.01.

The preceding data demonstrates that the number of suppressor cells needed to suppress the local expression of DTH in immunized mice is specifically amplified by immunization with the same antigen used to induce these cells. To determine durability and timing of the suppressive ability at the site where suppressor T cells were transferred, spleen cells recovered from donor mice that received an intracameral injection of TNP-BSA and immunization with TNP-BSA were injected into the footpads of mice immunized with TNP-BSA. The mice were challenged immediately with epicutaneous PCl or were challenged 3 (11 days P.I.) or 5 days (13 days P.I.) after the AC-SPL spleen cells were injected into the footpad. Footpad swelling in response to epicutaneous PCl was suppressed by 70% in mice challenged immediately after the injection of spleen cells. However, swelling was reduced only 30% in footpads challenged 3 days after the injection of the AC-SPL cells and was not suppressed in footpads challenged five days after the injection of the AC-SPL cells (Fig. 5A). However, footpad swelling of immunized mice challenged 11 or 13 days after immunization was suppressed by the injection of AC-SPL cells concomitant with epicutaneous challenge with PCl. We conclude that the lack of suppression of swelling by suppressor spleen cells “parked”at the challenge site for 3 days or 5 days was not due to a resistance to suppression in immune effector T cells 11 or 13 days after immunization. (Fig. 5B).

## Discussion

Cell-mediated regulation of an immune response is a complex system that includes CD4^+^, FoxP3^+^regulatory T cells (Treg), CD4^+^, FoxP3^ = ^ T cells, NKT cells and CD8^+^ T cells [1]. CD4^+^, FoxP3^+^ T cells are not obligatory for ACAID [22]. However,] CD4^+^, FoxP3^ = ^ T cells and NKT cells both participate in the induction of splenic CD8^+^ suppressor T cells after the intracameral injection of antigen [17], [20]. Immunoregulatory thymic NKT cells induced by an intracameral injection of antigen also emigrate from the thymus to the spleen. In the spleen, CD4^+^ T cells, peripheral NKT cells, recent NKT thymic immigrants, B cells, and the F4/80^+^ cells influenced by transit through the anterior chamber interact in the activation of CD8^+^ regulatory T cells that effect the suppression of effector Th1 cells [13]–[15], [20]–[22]. Antigen is required for the induction of CD8^+^ suppressor T cells by intracameral injection. Therefore, spleen cells recovered from naïve mice or mice receiving an intracameral injection of PBS only are not suppressive. In addition, AC-SPL cells recovered from mice receiving an intracameral injection of an antigen different from that used to immunize the mice do not suppress the expression of DTH to another antigen [9], [21], [23].

The suppression of antigen-induced swelling (expression of DTH) by AC-SPL cells is directly proportional to the number of AC-SPL cells introduced to the site challenged by antigen. In fact, the injection of AC-SPL cells intravenously at the time of challenge does not suppress the expression of DTH [9], [21]. Because of the direct, quantitative relationship of the number of AC-SPL cells required to suppress the expression of DTH, we derived a metric to relate the number of AC-SPL cells required to effect a 50% suppression of swelling (RSw50). WE reasoned that changes in the RSw50 would reflect a change in the number and/or activity of suppressor T cells in the AC-SPL cell population.

Since suppression of the expression of DTH in the LTS is due solely to CD8^+^ T cells, we titrated the suppressive activity of CD8^+^ AC-SPL cells in the LTS vs unseparated AC-SPL cells. The lower RSw50 of the CD8^+^ cell-enriched AC-SPL cells (85–92%) as compared to unseparated AC-SPL cells is consistent with the 33–35% of CD8^+^ cells in the latter population and substantiates the use of the LTS to provide for a quantitative estimate of CD8^+^ suppressor cell-mediated suppression. In the absence of a marker for CD8^+^ suppressor T cells, this assay does not provide for the enumeration of CD8^+^ suppressor T cells but does provide for a quantitative estimate of the *strength* of suppression similar to that of *in vitro* assays to measure cytotoxic T cells. However, the suppression in the LTS is not dependent on cell-mediated cytotoxicity [9].

The suppression of the expression of DTH in immunized mice by CD8^+^ AC-SPL suppressor T cells is dependent on TGF-β produced by these cells [21], [24] and is proportional to the number of AC-SPL cells injected into the site challenged with antigen. Because the suppression mediated by CD8^+^ suppressor T cells is not due to “bystander” suppression [9], suppression is likely mediated by cell-cell contact consistent with the dose-response relationships we observed between the reduction in DTH-induced swelling and the number of CD8^+^ suppressor spleen cells injected into the site in immunized mice challenged with antigen. Moreover, a single effector T cell can induce a DTH swelling reaction [25]. Therefore, very few CD8^+^ suppressor T cells would be required to suppress the initiation of DTH-induced swelling. As a result, these regulatory-effector cells suppress the Th1-mediated recruitment of monocytic cells associated with swelling [9].

The increase in suppressor cell activity (reduction in RSw50) induced by immunization with the same antigen as that injected into the anterior chamber is consistent with our observation that the most effective dose of antigen to induce the AC-SPL cells that suppress the expression of DTH is that which is optimal to induce DTH [23]. Moreover, the dose of antigen required to induce DTH or to expand the AC-SPL suppressor cells differs for BALB/c and C57BL/6 mice. Since the suppression elicited by the CD8^+^ suppressor cells requires interferon-γ, (IFN-γ [26], [27]), the induction of a Th1 response should foster the activity of these suppressor T cells. Perhaps the specific increase in CD8^+^ suppressor T cell-mediated suppression is due to a conversion of CD8^+^ effector T cells to a suppressive phenotype by TGF-β [10]. The total number of CD8^+^ cells does not increase in the spleen or lymph nodes after the intracameral injection of antigen and immunization [4], [28]. Overall the proportional increase in CD8^+^ cells is quite small, consistent with an antigen-specific increase in reactive clones. In other words, observing an increase in these regulatory cells is observing an antigen-induced increase. Taken together, the metric generated by the use of limiting numbers of antigen-specific regulatory T cells provides a method to quantitatively examine an immune response that generates these cells

By “parking” splenic CD8^+^ suppressor cells in the footpads of immunized mice we could estimate the stability of the cells or their function. Three-5 days after injection of these cells into the footpad there was no suppression of swelling induced by antigenic challenge. This could be due to the cells emigrating from the site and/or a loss of function. Since the DTH response is intact it is likely that the IFN-γ required for suppressive function is present. Presently, we are investigating the basis of the durability of the splenic regulatory T cells introduced to the site challenged by antigen. In summary, the expansion of an antigen-specific CD8^+^ regulatory T cell population appears to be a consequence of an immune response to the immunogen. The use of the quantitative LTS could provide information on the parameters required to expand this population as well as provides a method to quantify antigen-specific, cell-mediated immunoregulation.
